# Biology, Bias, or Both? The Contribution of Sex and Gender to the Disparity in Cardiovascular Outcomes Between Women and Men

**DOI:** 10.1007/s11883-022-01046-2

**Published:** 2022-07-01

**Authors:** Sarah Gauci, Susie Cartledge, Julie Redfern, Robyn Gallagher, Rachel Huxley, Crystal Man Ying Lee, Amy Vassallo, Adrienne O’Neil

**Affiliations:** 1 Institute for Mental and Physical Health and Clinical Translation, Deakin University, Geelong, VIC Australia; 2 School of Public Health and Preventive Medicine, Monash University, Melbourne, VIC Australia; 3 Susan Wakil School of Nursing and Midwifery, University of Sydney, Sydney, NSW Australia; 4 School of Health Sciences, University of Sydney, Sydney, NSW Australia; 5 Faculty of Health, Deakin University, Melbourne, VIC Australia; 6 School of Population Health, Curtin University, Perth, WA Australia; 7 The George Institute for Global Health, University of New South Wales, Sydney, NSW Australia

**Keywords:** Gender, Cardiovascular disease, Sex, Women

## Abstract

**Purpose of Review:**

Cardiovascular disease (CVD) is the leading cause of mortality and morbidity worldwide for both men and women. However, CVD is understudied, underdiagnosed, and undertreated in women. This bias has resulted in women being disproportionately affected by CVD when compared to men. The aim of this narrative review is to explore the contribution of sex and gender on CVD outcomes in men and women and offer recommendations for researchers and clinicians.

**Recent Findings:**

Evidence demonstrates that there are sex differences (e.g., menopause and pregnancy complications) and gender differences (e.g., socialization of gender) that contribute to the inequality in risk, presentation, and treatment of CVD in women.

**Summary:**

To start addressing the CVD issues that disproportionately impact women, it is essential that these sex and gender differences are addressed through educating health care professionals on gender bias; offering patient-centered care and programs tailored to women’s needs; and conducting inclusive health research.

## Introduction

Cardiovascular disease (CVD) has historically been perceived as a male disease; however, it is the leading cause of mortality and morbidity worldwide for both men and women [1]. Despite this, CVD is understudied, underdiagnosed, and undertreated in women [2]. Both sex — biological and anatomical factors — and gender — cultural and societal factors — have been found to be important modifiers of CVD but are often underappreciated in clinical practice. This leads to inequality in the outcomes and treatment of women with CVD. For example, mortality rates are improving at a faster rate in men compared to women [3]. Women also experience a greater delay in emergency response times [4], diagnosis, and revascularization compared to men [5, 6]. These delays are thought to be due to biological differences in the presentation of CVD in women and gender biases in both patients and health care professionals [7, 8]. There is growing recognition for the need to address issues with sex and gender in health research. However, much remains to be done [9–12].

The aim of this narrative review is to explore the complex sex and gender differences observed in the research, treatments, and outcomes of women with CVD. The review also includes recommendations for research and practice. For context, it is important to distinguish and define both sex and gender. Sex refers to the variation and expression of biological attributes such as genetics (e.g., sex chromosomes), sex hormones, anatomy, and physiology [13, 14]. In comparison, gender is a multidimensional construct that has been developed over time from social, cultural, and behavioral factors. Gender incorporates the dimensions identity, societal roles, interpersonal relationships, and institutionalized gender norms [13].

## Examples of Sex-Specific Risk Factors of CVD

There are well established biological sex differences in the risk factors for CVD [15, 16]. Reviews of the literature demonstrate that sex-specific risk factors include age of first menarche, menopause, reproductive endocrine disorders, and pregnancy complications [17–20]. However, even shared risk factors such as diabetes and smoking have been found to impact CVD risk differently in women. Meta-analyses have demonstrated that the relative risk (RR) of CVD is 44% and 25% higher in women with diabetes and women who smoke when compared to men (RR ratio 1.44 (95% CI 1.27 to 1.63) and 1.25 (95% CI 1.12 to 1.39)) [21, 22], whereas blood pressure, body mass index, and hyperlipidaemia have been found to have a similar impact on CVD risk in men and women [22–24]. There are also differences in the presentation of CVD symptoms. While chest pain is the most commonly reported symptom in both men and women, women often have different or asymptomatic presentations compared to men [25]. Women regularly report additional symptoms such as epigastric symptoms, palpitations, and shortness of breath [25]. While understanding sex differences in CVD risk is important, it only provides part of the story. Just as there are differences in biological risk factors, there are differences in sociocultural risk factors between different genders [15, 26, 27].

## Examples of Gender-Specific Risk Factors for CVD

Gender-specific risk factors include environmental factors that, for non-biological reasons, contribute to health disparities like exposure to violence, sociocultural behaviors and attitudes, and socioeconomic barriers [11, 28, 29]. A review article by O’Neil et al. [26] describes how gender acts as a social determinant of CVD. O’Neil et al. describe how the adoption of health behaviors is highly gendered. For example, the adoption of certain health behaviors such as being active and playing sports is encouraged in young men more so than in young women [30, 31]. There have been successful school-based intervention that have increased physical activity and reduced risk factors such as obesity in women [32]. These findings demonstrate that some of the gender-specific risk factors can be modified.

Another review conducted by Connelly et al. [13] describes how gender identity (e.g., personality traits and psychosocial stress), gender roles (e.g., carer responsibilities and primary earner status), and gender relations (e.g., marital status) increased CVD risk for women. Further, interpersonal relationships can also impact the risk of CVD. For instance, women who experience intimate partner violence and domestic abuse have an increased risk of CVD (incidence rate ratio 1.44 (95% CI, 1.24–1.6)) [33, 34]. In addition, women who live alone report greater barriers to care, such as greater financial issues in the recovery after acute coronary syndrome [35], which may impact their risk of a secondary event. Sexism has also been proposed as a psychosocial factor that influences the risk of CVD [36]. For example, the experience of sexism has been related to increased alcohol consumption and smoking in women [37], behaviors that increase the risk of CVD.

Gender biases in medical professionals also contribute to the differences in outcomes for CVD between women and men. An example of this gender bias across health outcomes is demonstrated when examining the association between patient-physician gender concordance and patient outcomes. Women have a higher mortality rate and worse outcomes when treated by male doctors [8]. While this may also be due to bias in reporting by women, these outcomes are improved when women have a female doctor or if their male doctor works with women and has treated more women in the past [8]. These biases are also present in the primary health care setting, women are less likely than men to have their CVD risk [38] and smoking status assessed [39]. After treatment for a cardiac event, there are gender biases that impact who bears the burden of care during recovery; in general, women tend to take on caregiving roles within families [40]. This bias may affect to whom information about care and recovery is directed to, for example, in heterosexual couples, the responsibility of care may be placed on the woman. In the care of cancer patients, this burden of care results in women reporting worse mental health outcomes [41, 42]. There is also bias in referral to secondary prevention programs. A meta-analysis conducted in 2015 found that men were 1.5 times more likely to be referred to cardiac rehabilitation (CR) than women (odds ratio 0.68 (95% CI 0.62 to 0.74)) [43]. Once referred to CR, women are also less likely to attend [44, 45•]. This is due to several barriers, including carer responsibilities, work commitments, geographical barriers, and perceptions about program characteristics [46].

## Examples of Intersecting Sex and Gendered Risk Factors for CVD

**Fig. 1:**
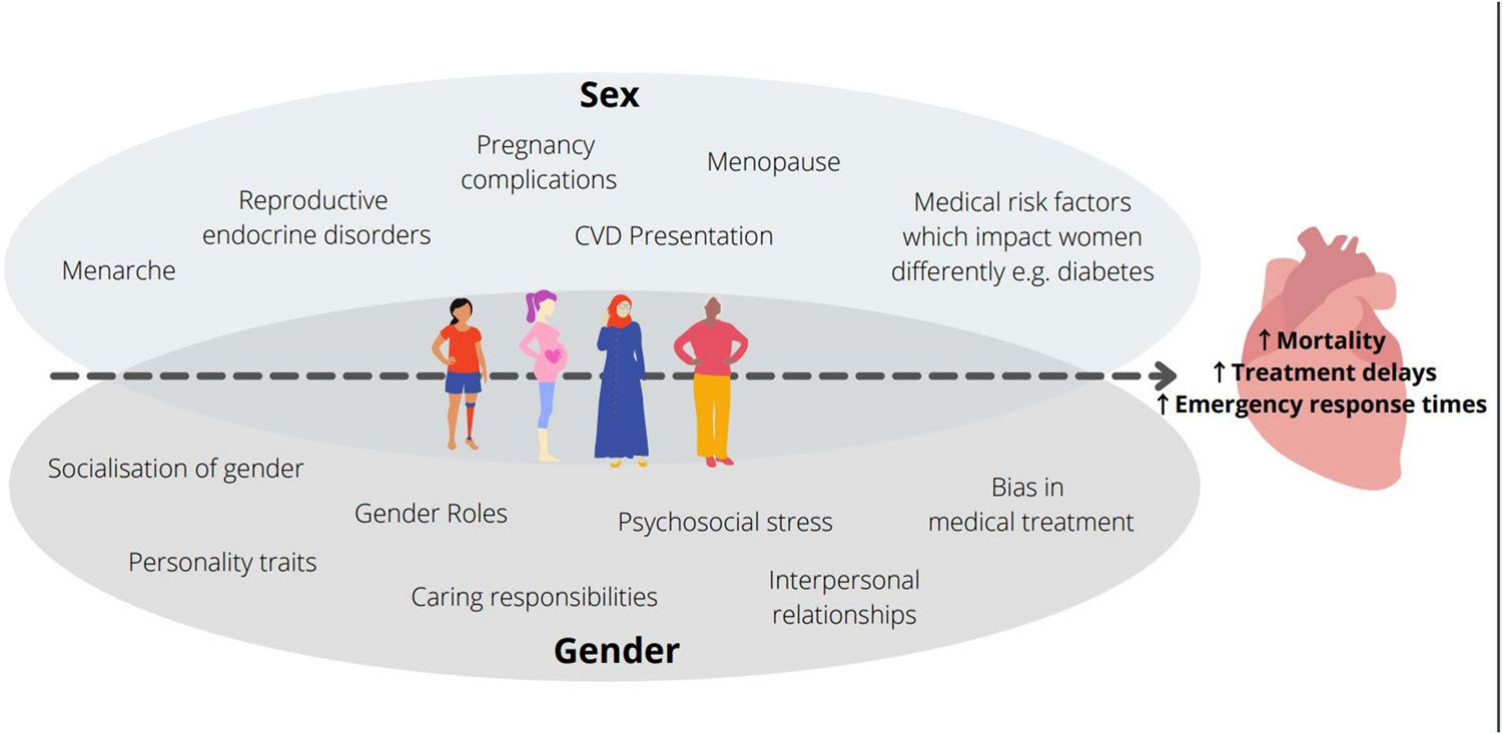
Examples of the contribution of both sex and gender across the life course on CVD outcomes in women

It is difficult to disentangle sex and gender influences on the differences in diagnosis, treatment, and outcomes for women with CVD. This is because both gender and sex interact to contribute to this discrepancy (Fig. 1). For example, psychosocial and biological factors increase the risk of mental health issues which in turn have been found to increase the risk of CVD [47]. Overall, these mental health issues disproportionately affect women more than men [48] and have been found to be an independent risk factor for CVD in women [49]. The diagnosis of depression has also been associated with worse outcomes, including morbidity and mortality for people with CVD [50, 51].

Sex and gender also interact to impact how accurately women interpret their CVD risk [5], resulting in men being more likely to interpret their symptoms as cardio specific during myocardial infarction (51.7 vs. 46.0%; *p* = 0.02) [52]. This misinterpretation of symptoms has been found to result in a 2-h delay in seeking treatment [52]. Qualitative research has also demonstrated how sex and gender interact to impact the delay in treatment for women, the interpretation of risk in combination with symptom presentation, and past responses from health professionals impact the time women take to seek treatment during their first cardiovascular event [53]. On average, women with ST-elevation myocardial infarction (STEMI) have a 30-min longer ischemic time when compared to men after controlling for confounders [5]. After further investigation, there are delays in both symptoms to door time (time taken from the presentation of symptoms to arrival at hospital) and door to balloon time (time from arrival to revascularization) for women [5].

These delays seem to be caused by multiple factors, highlighting complex interactions between sex and gender. As established, women take longer to seek medical treatment (on average 3.2 h for women versus 2.4 for men) [25]. There are also delays in the time it takes for women to arrive at the hospital. In Norway, women with STEMI were given lower priority for ambulance services than men with similar presentations and took longer to arrive at the hospital [4]. Once women arrive at the hospital, there is also a delay in receiving the correct diagnosis and intervention [54]. These delays in receiving life-saving care are associated with a higher mortality rate in women [5]. An Australian study found similar results with women being 18% less likely to receive an urgent care allocation upon admission to the emergency department (OR 0.82 (95% CI 0.79 to 0.85)), 16% less likely to be seen by an emergency physician in the first hour of arrival at emergency (mean difference 0.15 (95% CI 0.13 to 0.1)), 20% less likely to have a diagnostic troponin test (OR 0.80 (95% CI 0.77 to 0.83)), 36% less likely to be admitted to a special care unit (OR 0.64 (95% CI 0.61 to 0.68)), and finally, women were also found to be more likely to die during their hospital admission [55••].

## Recommendations for Researchers

Historically, women have been underrepresented in health research, including CVD research [2, 9, 56]. This underrepresentation of women in research partially explains the incomplete understanding of CVD symptomology and presentation in women. For example, the 2016 National Heart Foundation of Australia & Cardiac Society of Australia and New Zealand clinical guidelines for the management of acute coronary syndromes was based on research that had inequality in sex and gender representation [57]. Scovellsce et al. (2020) examined the studies used to inform the guidelines and found that beyond mere participation of women in research, only 70% of studies mention either sex or gender in the body of the publication; 78% of studies included reported on the number of men and women in the study cohort; only 50% and 18% of studies reported on sex or gender disaggregated results respectively in the exposure and primary outcome; and only 23% included sex or gender in the statistical model. These findings highlight the gender bias in evidence underpinning current CVD guidelines and the need for policies to ensure that women are equally represented in all health research, including CVD.

While progress has been made to ensure the inclusion of women in research and the consideration of sex in the analysis of health outcomes, there is still need to consider gender in the analysis and interpretation of health outcomes [19, 58•]. Table 1 describes some of the recommendations for how researchers can do this.

**Table 1:** Recommendations for researchers

Recommendation	Reason	Examples
Include both sex and gender-related variables	• Women are underrepresented in CVD research [2] • Sex and gender contribute to risk • Sex is a poor proxy for gender. It fails to account for social, political, and economic factors that may impact health outcomes [9]	• Include policies to ensure researchers account for sex as a biological variable to receive funding [59] • Collect sex and gender data [60] • Collect social and economic data as additional variables that may impact differences between men and women and how these factors impact health outcomes [9, 61–63] • Include intersectional analysis as it goes beyond investigating how different factors contribute to risk but at how these factors interact to impact risk [64, 65]
Include gender diverse populations in research	• Risk factors for CVD have been found to impact transgender and gender non-conforming populations differently • Gender minority groups have higher CVD risk when compared to cisgender adults [66]	• Accurately collect and categorize sex and gender data [67]
Use correct definitions	• Sex and gender are frequently used interchangeably and incorrectly in research [12, 19]	• Considered efforts should be made to correctly define and include sex and gender in health research [13, 68] • Provide clear definitions and distinction in research outputs as to whether talking about a biological difference or a gender difference
Tools to aid researchers		
Instruments and checklists	• Gender awakening [69] tool is a checklist to determine if all sex and gender considerations have been included in the research • Gendered innovations also include a checklist that can be used to ensure that both gender and sex have been included in all stages of the research process [70] • The “Sex and gender in systematic reviews: Planning tool” has been developed to help reviewers make sure they are asking and answering any sex and gender-based differences in their systematic reviews [71] • Gender as a Sociocultural Variable (GASV) is a questionnaire that was designed to assess specific gender-related behaviors and attitudes that contribute to health outcomes [11] • The Toolkit Gender in EU Funded Research provides guidance to researchers when conducting sex and gender-sensitive research [72]	
Policies	• Canadian Institutes of Health Research (CIHR) policy to use sex and gender-based analysis in health research [73] • The National Institutes of Health Policy on Sex as a Biological Variable [74] and on the Inclusion of Women and Minorities as Subjects in Clinical Research [75] • The Sex and Gender Sensitive Research Call to Action Group published recommendations for Australian stakeholders [58•]. They include suggestions that Universities and training institutes develop curricula that recognize the sex and gender differences in health and that professional societies, government bodies, research funders, and journals establish policies to ensure inclusion of sex and gender in research [58•]	
Analysis	• Sex and or gender-based analysis [9, 61] as fundamental throughout the research process, at a minimum, results should be sex disaggregated, that is analyzed separately [76]	

## Recommendations and Clinicians

Based on the persistent sex and gender differences in the risk, diagnosis, and treatment of CVD, several steps must be taken to ensure no group of people are disproportionately affected by CVD. A recent scoping review demonstrated that there are 13 published studies that evaluate interventions aimed at reducing the gender disparity CVD outcomes in health care [77]. This highlights the need for additional evidence-based interventions to address the sex and gender bias in health care. Table 2 describes some of the ways clinician can address these issues.

**Table 2:** Recommendations for clinicians

Recommendation	Reason	Example
Addressing bias in practitioners	• Delays in diagnosis and treatment for women [5] • Differences in outcomes for women [55••]	• Practitioners and health professionals should engage in training about gender bias and the sex and gender-specific differences in the presentation and treatment of CVD [19] • Introduce protocols and programs to reduce gender disparities in diagnosis and treatment [78, 79]
Addressing bias in patients	• Women are less likely to accurately assess their risk of CVD • Women take longer to call emergency services when having a MI [25]	• Improve public health communication and education about how myocardial infarction symptoms differ between women and men [16]. This will allow women to understand the symptoms they need to be aware of and help them receive timely care [19]
Patient-centered care	• Women experience greater barriers to care	• Incorporating patient-centered care will help address some barriers women face, by informing and engaging women in their care. It has been found to positively improve health experiences and outcomes [80] • Women-focused programs [81] and women-focused cardiac rehabilitation are programs tailored to the needs and interests of women [82, 83]

## Conclusions

There are sex and gender differences in the risk, presentation, treatment, and research of CVD. These differences have resulted in women being disproportionally affected by CVD. While some differences are due to biological differences between males and females, many can be attributed to biases in both patients, health professionals, and societal norms. Further, sex and gender appear to interact to contribute to the differences in risk and outcomes for women. In order to reduce the inequity in health outcomes for women, these biases must be addressed through improving the communication of biological differences in the presentation of CVD; educating health care professionals on gender bias; offering patient-centered care and programs tailored to women’s needs; and conducting research that includes sex and gender-based analysis. By conducting more inclusive research and addressing the gender biases present in health care, we may start addressing the CVD issues that disproportionately impact half of the world’s population.
